# Electroformation of Giant Unilamellar Vesicles from Damp Lipid Films with a Focus on Vesicles with High Cholesterol Content

**DOI:** 10.3390/membranes14040079

**Published:** 2024-03-27

**Authors:** Ivan Mardešić, Zvonimir Boban, Marija Raguz

**Affiliations:** 1 Department of Medical Physics and Biophysics, University of Split School of Medicine, 21000 Split, Croatia; imardesi@mefst.hr (I.M.); zvonimir.boban@mefst.hr (Z.B.); 2 Doctoral Study of Biophysics, Faculty of Science, University of Split, 21000 Split, Croatia

**Keywords:** GUV, electroformation, cholesterol, damp lipid film, lipid film deposition, cholesterol demixing artifact

## Abstract

Giant unilamellar vesicles (GUVs) are membrane models used to study membrane properties. Electroformation is one of the methods used to produce GUVs. During electroformation protocol, dry lipid film is formed. The drying of the lipid film induces the cholesterol (Chol) demixing artifact, in which Chol forms anhydrous crystals which do not participate in the formation of vesicles. This leads to a lower Chol concentration in the vesicle bilayers compared to the Chol concentration in the initial lipid solution. To address this problem, we propose a novel electroformation protocol that includes rapid solvent exchange (RSE), plasma cleaning, and spin-coating methods to produce GUVs. We tested the protocol, focusing on vesicles with a high Chol content using different spin-coating durations and vesicle type deposition. Additionally, we compared the novel protocol using completely dry lipid film. The optimal spin-coating duration for vesicles created from the phosphatidylcholine/Chol mixture was 30 s. Multilamellar vesicles (MLVs), large unilamellar vesicles (LUVs) obtained by the extrusion of MLVs through 100 nm membrane pores and LUVs obtained by extrusion of previously obtained LUVs through 50 nm membrane pores, were deposited on an electrode for 1.5/1 Chol/phosphatidylcholine (POPC) lipid mixture, and the results were compared. Electroformation using all three deposited vesicle types resulted in a high GUV yield, but the deposition of LUVs obtained by the extrusion of MLVs through 100 nm membrane pores provided the most reproducible results. Using the deposition of these LUVs, we produced high yield GUVs for six different Chol concentrations (from 0% to 71.4%). Using a protocol that included dry lipid film GUVs resulted in lower yields and induced the Chol demixing artifact, proving that the lipid film should never be subjected to drying when the Chol content is high.

## 1. Introduction

Vesicles are membrane models consisting of one or more lipid bilayers filled with aqueous solution. They are commonly used to investigate membrane properties under controlled conditions [1,2]. Depending on their structure, lipid vesicles can be unilamellar, multilamellar, or oligolamellar. Unilamellar vesicles have only a single lipid bilayer, multilamellar vesicles (MLVs) contain multiple lipid bilayers arranged in concentric circles, and oligolamellar vesicles contain smaller vesicles within the outer bilayer. With respect to their size, unilamellar vesicles are usually divided into three groups: small (SUVs) (<100 nm), large (LUVs) (100 nm–1 μm), and giant (GUVs) (>1 μm). SUVs and LUVs are the most common type of unilamellar vesicles produced by extrusion, and they are often used for drug delivery studies or in protocols for the formation of GUVs [3] or supported lipid bilayers [4]. Among these three groups, researchers studying membrane properties and organization are most interested in GUVs, as their size is similar to that of eukaryotic cells.

The first attempt to form GUVs was performed by Reeves and Dowben [2]. In their approach, the lipid mixture is deposited onto the electrode, dried to form a dry lipid film that is then rehydrated, and the aqueous solution is driven between the lipid stacks due to osmotic pressure (gentle hydration). Because of the amphiphilic nature of lipids, it is unfavorable for their hydrophobic acyl chains to be exposed to the aqueous solution, so the lipid bilayers enclose, forming vesicles. Even though this method is straightforward and simple, it results in a low GUVs yield, with many defects [5].

One of the most commonly common currently used methods for the production of GUVs is electroformation, which was developed by Angelova and Dimitrov in 1986 [6]. This method improved the previous approach by applying an external electric field to the lipid film, in addition to the use of hydration. In their protocol, lipids are dissolved in an organic solvent and deposited on an electrode. The organic solvent is then evaporated using a stream of inert gas and a vacuum. The electrode with the lipid film is used to construct an electroformation chamber, which is filled with a desired aqueous solution and connected to an external alternating electric field. The electric field and osmotic pressure promote the detachment of the lipids from the electrode, leading to the formation of GUVs [7]. Compared to samples obtained using the gentle hydration method, the vesicles formed by electroformation exhibit a higher yield, a lower number of defects, and a higher proportion of unilamellar vesicles [5].

The electroformation method has evolved significantly since its inception, with various modifications made to the tested protocols [8,9,10] in order to improve the method’s reproducibility and enable its compatibility with a wider range of lipid mixtures. Some groups attempted to improve the method by replacing the lipids dissolved in an organic solvent with an aqueous solution of SUVs or LUVs [11]. It was concluded that the use of a unilamellar vesicle aqueous solution improved the efficiency of GUVs formation compared to the deposition of lipids dissolved in an organic solvent. It was also shown that the formation of GUVs, with buffers as internal solutions, is easier when using buffer-loaded SUVs or LUVs compared to the previous approach, in which the buffer would be applied to a dry lipid film [11]. Another advantage of this modification is the possibility of improved proteoliposome formation, due to reduced protein denaturation when the organic solvent is removed from the protocol [11,12,13].

Another issue with the traditional protocol was the use of the drop-deposition method for lipid film deposition [6,7,9]. The problem with this approach is the formation of lipid films of nonuniform thickness, resulting in high vesicle heterogeneity in the sample and low experimental reproducibility. Various alternative approaches have been tested to address this issue [14,15,16]. The one we found to be optimal in terms of ease of use and quality of the final result is the spin-coating technique, in which uniform lipid films are obtained by depositing the lipid solution onto a flat surface and spinning it at a high angular velocity [15,17,18,19,20].

The initial electroformation protocol also turned out to be incompatible with lipid mixtures containing a high cholesterol (Chol) concentration. This is due to the precipitation of Chol into anhydrous Chol crystals during the lipid film drying phase [21]. When the film is rehydrated, these anhydrous Chol crystals do not participate in the bilayer formation, resulting in a lower Chol concentration in the vesicle bilayers compared to the Chol concentration in the initial lipid solution. Chol demixing can be avoided by using the rapid solvent exchange (RSE) method [22,23]. In this method, lipids dissolved in an organic solvent are first mixed with an aqueous solution. By suddenly decreasing the pressure, the organic solvent is evaporated, leaving behind an aqueous solution of MLVs. In order to avoid Chol demixing artifact during GUVs preparation, Baykal-Caglar et al. tested the use of electroformation from a damp lipid film formed by depositing MLVs, produced using the RSE method, onto the electrode [3]. The final results were positive, indicating a higher Chol content in the formed GUVs compared to that of the GUVs obtained using the original protocol. However, the described protocol is very time consuming, due to the long lipid film drying time (22–25 h), and the MLVs were deposited using the drop-deposition method, inevitably resulting in a non-uniform film.

In addition to the deposition techniques and the properties of the lipid mixtures, the cleanliness of the electrodes has also been shown to influence the electroformation results. Pretreatment of the electrodes with plasma improved the efficiency of the formation of GUVs containing buffers with physiological charge levels. This is probably due to the fact that the plasma makes the electrode surface more hydrophilic, facilitating the hydration of the lipid film and the subsequent formation of the lipid bilayers [24].

**Figure 1:**
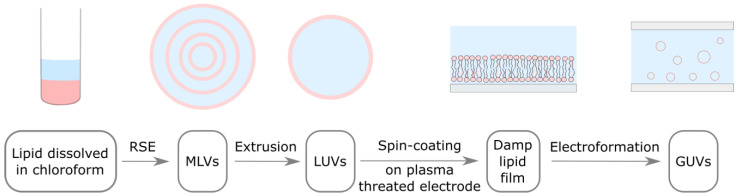
Adapted electroformation protocol. Lipids dissolved in organic solvent (pink) are mixed with an aqueous solution (light blue). MLVs are formed using the RSE method. MLV solution is extruded through a filter with membrane pores in order to obtain LUVs. LUV solution is spin-coated on a plasma threated electrode, where these vesicle rupture and form a damp lipid film. An electrode with damp lipid film is used to build an electroformation chamber, where lipid bilayers detach and form GUVs under the influence of osmotic pressure and an alternating electric field.

In our previous study, we adapted the traditional electroformation protocol to incorporate all of the aforementioned improvements, allowing us to bypass the dry lipid film phase and produce a uniform damp lipid film (Figure 1) [25]. Inspired by the vesicle fusion method for the preparation of supported lipid bilayers [4,26,27], the adapted protocol uses spin-coating to deposit an aqueous solution of LUVs onto a plasma-treated electrode. The hydrophilized surface induces LUV rupturing and the formation of lipid bilayers on the electrode surface. The bilayers later detach and form GUVs under the influence of osmotic pressure and an alternating electric field. With respect to the approach of Baykal-Caglar et al., these modifications significantly shorten the duration of the protocol, while also increasing the experiment reproducibility. In order to make the protocol compatible with high Chol concentrations, the deposited LUVs were obtained by extruding a solution of MLVs, produced using the RSE method, and the lipid film was not dried after spin-coating.

The efficiency of the protocol was tested using 1/1 Chol/1-palmitoyl-2-oleoyl-glycero-3-phosphocholine (POPC) and 1/1/1 Chol/POPC/sphingomyelin lipid mixtures [25]. This ternary mixture was selected because these three lipid types are highly prevalent in biological membranes. Particularly noteworthy is the phenomenon of liquid order–liquid disorder phase separation, which plays a crucial role in the development of lipid rafts [28,29,30,31]. Furthermore, recent research has also shown the possibility of the coexistence of three phases of similar composition in GUVs [32].

Compared to the protocol involving a drying step, the new protocol resulted in a similar or better GUV yield, while exhibiting the potential to significantly reduce the Chol demixing artifact [25]. In order to further increase the reproducibility and yield of the obtained samples, this study will focus on the further optimization of the new protocol.

Special focus is placed on GUVs with a high Chol content, for which the Chol demixing issue is more pronounced. Chol plays diverse roles in phospholipid membranes, impacting membrane thickness and rigidity [33,34,35], domain formation [36,37], cell signaling [38], and bending modulus [39]. Positioned vertically within the phospholipid bilayer, its polar head aligns with other phospholipid heads, while its fused ring structure resides within the acyl chain region. This orientation enables cholesterol to modulate the lateral organization of phospholipids, affecting membrane properties across various depths. These regulatory actions include changes in the bilayer transition temperature [40] and the emergence of specific membrane phases and domains. For vesicles with a Chol content lower than ~15 mol%, the lipid bilayer is in a liquid disordered phase. At 30–50 mol%, it is in a liquid ordered phase. Cholesterol bilayer domains (CBDs), containing only Chol molecules with no phospholipids, start forming when the Chol content is greater than 50 mol%. After reaching the Chol solubility limit at ~66 mol%, excess Chol precipitates in the form of Chol crystals [41].

Modeling membranes with such a high Chol concentration is important for groups such as ours that study the properties of the eye lens plasma membranes [21,42,43] or for groups performing atherosclerosis research [44]. The protocol may also be beneficial for the formation of proteoliposomes by reducing the denaturation of proteins that occurs during the preparation of GUVs from lipids dissolved in an organic solvent or during film drying.

## 2. Materials and Methods

### 2.1. Materials

POPC and Chol were purchased from Avanti Polar Lipids Inc. (Alabaster, AL, USA), while the fluorescent dye 1,1-dioctadecyl-3,3,3,3-tetramethylindocarbocyanine Perchlorate (DiIC_18_(3)) was acquired from Invitrogen, Thermo Fisher Scientific (Waltham, MA, USA). Lipids were stored at −20 °C when not in use. Indium tin oxide (ITO) coated glass (ICG-90 INS 115, resistance 70–100 Ω), measuring 25 × 75 × 1.1 mm, was procured from Delta Technologies (Loveland, LO, USA). Fresh ITO-coated glass was employed for each preparation to ensure the efficient formation of GUVs [45]. Mili-Q deionized water (Merck, Rahway, NJ, USA), preheated to 60 °C, served as the internal chamber solution.

### 2.2. Preparation of Multilamellar Vesicles Using the Rapid Solvent Exchange Method

Initially, multilamellar vesicles (MLVs) were generated using a home-built RSE device to circumvent the cholesterol demixing issue. A lipid mixture dissolved in chloroform was prepared by blending 25 mg/mL of POPC, 20 mg/mL of cholesterol (Chol), and 1 mg/mL of DiIC_18_(3), in appropriate proportions. The Chol/POPC mixing ratios were maintained between 0 and 2.5 (Chol mixing concentration of 0% to 71.4%), while the DiIC_18_(3)/POPC mixing ratio was fixed at 1/500. The total lipid mass amounted to 2.1 mg. Subsequently, 400 µL of Mili-Q water was introduced into the solution, and the mixture was vortexed (Vortex IR, Star Lab, Blakelands, UK) at an angular velocity of 2200 rpm. Throughout the vortexing process, the pressure was regulated to approximately 0.05 bar using a vacuum pump (HiScroll 6, Pfeiffer Vacuum, Asslar, Germany). The sample was maintained at the specified pressure for 90 s to ensure the complete removal of residual organic solvent.

### 2.3. Preparation of Large Unilamellar Vesicles

The MLVs solution was extruded utilizing an Avanti Mini Extruder (Avanti Polar Lipids, Inc, Alabaster, AL, USA), passing through either a 50 or a 100 nm pore diameter polycarbonate filter (Nuclepore Track-Etch Membrane, Whatman, UK) 15 times to achieve a uniform LUV suspension. To prevent the loss of the lipid suspension, the filters and membranes were pre-wetted with Mili-Q water. Additional Mili-Q water was added to the LUV solution to reach a lipid concentration of 3.5 mg/mL.

### 2.4. Preparation of the Damp Lipid Film

Prior to conducting the experiments, the ITO-coated glass was submerged in Mili-Q deionized water. Subsequently, the glass was wiped using lint-free cloths saturated with 70% ethanol. Following this, the glass underwent treatment with oxygen plasma for a duration of 1 min utilizing a plasma cleaner (PDC-002-HPCE with the PLASMAFLO PDC-FMG-2 attachment, Harrick Plasma, Ithaca, NY, USA) connected to a vacuum pump (HiScroll 6, Pfeiffer Vacuum, Assler, Germany).

Unless specified otherwise, a volume of 550 µL of LUV suspension was applied onto the hydrophilic plasma-treated ITO-coated glass electrode and promptly spin-coated using a spin-coater (SM-150, Sawatec, Sax, Switzerland). The electrode was spun at 600 rpm, achieving the final speed within 1 s. The duration of spin-coating, unless specified otherwise, was maintained at 30 s to guarantee the formation of a damp, uniform lipid film. To prevent unintended evaporation, the coated ITO-coated glass was transferred into a Petri dish and promptly utilized to form an electroformation chamber.

### 2.5. Electroformation Protocol

The electroformation chamber was constructed by sandwiching two 25 × 37.5 mm ITO-coated glasses with a 1.6 mm thick Teflon spacer in between. The electrodes were created by slicing a 25 × 75 mm ITO-coated glass using a diamond pen cutter. To form the electroformation chamber, the lipid-coated glass was sealed to the Teflon spacer with vacuum grease. Following the addition of 280 µL of Mili-Q water, the stopper was sealed using vacuum grease, ensuring that there was no contact between the grease and water to prevent any detrimental effects of grease contamination on GUV formation [9]. The chamber was secured with clamps at three points along the electrodes, two adjacent to the stopper and one opposite it. Subsequently, the chamber was connected to a pulse generator (UTG9005C, UNI–T, Dongguan City, China or PSG 9080, Joy-IT, Neukirchen-Vluyn, Germany) and placed in an incubator set to a temperature of 60 °C. Copper tape was applied to the outer edges of the electrode to enhance wire–electrode contact. Consistent with previous experiments [17,18,25], a voltage of 2 V and a frequency of 10 Hz were maintained. After 2 h, the pulse generator was turned off, and the chamber remained in the incubator for an additional hour.

### 2.6. Dynamic Light Scattering

Dynamic light scattering (DLS) was utilized to determine the hydrodynamic diameter and polydispersity index of the liposome suspensions (Litesizer 500, Anton Paar, Graz, Austria).

### 2.7. Fluorescence Imaging and Data Analysis

To cover the entire volume of the chamber, images were captured from 16 different regions of the sample. A total of 100 vesicles were then randomly selected from these images. In cases where the images did not contain 100 vesicles, all observed vesicles were included in the analysis. Imaging was conducted utilizing a fluorescence microscope (Olympus BX51, Olympus, Tokyo, Japan), and the vesicle diameters were measured using the line tool within Fiji software [46].

Unless specifically indicated otherwise, the numerical results are presented as the mean ± standard deviation. All data analysis and visualization were carried out using the R programming language [47].

## 3. Results and Discussion

In order to further improve our newly developed protocol, we performed experiments to test the effect of using different vesicle types during lipid film deposition, the effect of spin-coating duration, and the effect of different Chol concentrations. To confirm the utility of the optimized protocol, our samples were also compared to those obtained from fully dried lipid films.

### 3.1. Optimization of the Protocol

#### 3.1.1. The Spin-Coating Duration for Formation of Damp Lipid Film

We tested various coating durations ranging from 15 s to 60 s at an angular speed of 600 rpm. The deposited liquid was an aqueous suspension of LUVs obtained by extruding MLVs through a 100 nm pore diameter filter. We concluded that 30 s is the optimal duration when using the aforementioned lipid mixtures. A shorter duration of spin-coating resulted in an uneven film thickness or a film that was too wet, leading to the low reproducibility of the experiment and high GUV heterogeneity within the successful samples. As shown in our previous study, for a spin-coating duration of 30 s, the lipid film remained damp, with high GUV electroformation yield [25]. At a duration of 30 s, water-dissolved lipid droplets can still be seen on the electrode, which assures us that the lipid film is still damp. Longer spin-coating durations were avoided due to the risk of excessive drying of the lipid film and the induction of the Chol demixing artifact. To prevent additional drying of the film due to water evaporation, the electrode was immediately used to build an electroformation chamber.

#### 3.1.2. Comparison of MLVs and LUVs Deposition on the Electrode

Since the film in our protocol is kept damp, we rely on the fact that the vesicles to rupture when they come into contact with a hydrophilized electrode surface to obtain a lipid film [26,48,49]. However, how easily the vesicles rupture depends strongly upon the vesicle type and size [50]. Therefore, we compared the GUVs prepared from lipid films obtained with three different types of vesicles—MLVs formed using the RSE method, LUVs obtained by the extrusion of MLVs through 100 nm membrane pores (LUV 100), and LUVs formed by the additional extrusion of LUV 100 vesicles through 50 nm membrane pores (LUV 50). The Chol/POPC mixing ratio was maintained at 1.5 in all these experiments. DLS was used to determine the size distribution of the suspensions of each vesicle type. The average hydrodynamic diameter and polydispersity index were 761.1 nm and 0.29 for MLVs, 141.9 nm and 0.1 for LUV 100 vesicles, and 96.2 nm, and 0.12 for LUV 50 vesicles, respectively.

**Figure 2:**
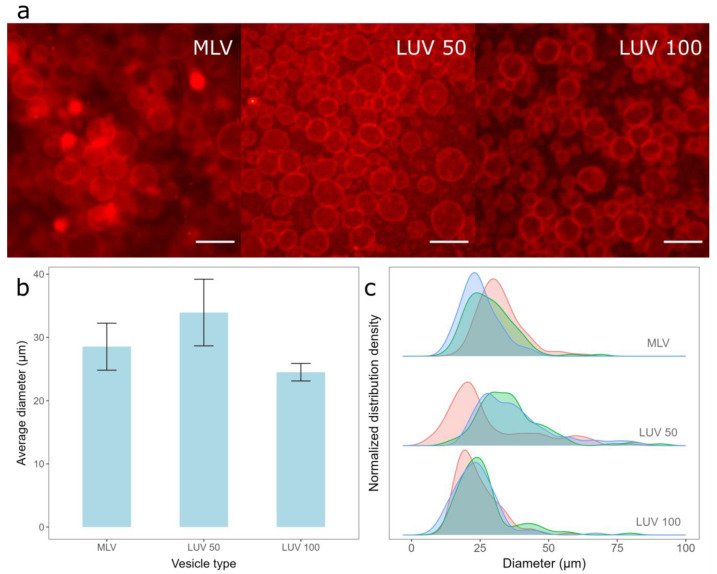
Electroformation of GUVs for deposition of different vesicle types. (**a**) Fluorescence microscopic images of GUVs formed when different vesicle types were deposited. MLV represents the deposition of MLVs on the electrode, LUV 100 represents the deposition of LUVs formed by extruding MLVs through 100 nm membrane pores, and LUV 50 represents the deposition of LUVs formed by extruding LUV 100 vesicles through 50 nm membrane pores. The scale bar represents 50 µm. (**b**) Comparison of average diameters and standard deviations of GUVs for preparations from three different vesicle types. The averages and standard deviations were calculated by averaging the mean diameters from three independent samples for each condition. (**c**) Size distribution densities of GUVs for deposition of different vesicle types for each sample. Each distribution density represents one independent sample (100 vesicles).

The deposition of the lipid film from all three vesicle types resulted in a high GUV yield (Figure 2a), with average diameters and standard deviations when using MLVs, LUV 50, and LUV 100 vesicles being 28.5 ± 3.71 µm, 33.9 ± 5.27 µm, and 24.5 ± 1.39 µm, respectively (Figure 2b). Although the average diameter of the GUVs obtained from LUV 100 vesicles deposition was the smallest, this method resulted in the highest reproducibility among the samples (smallest error bars in Figure 2b). In addition, the widths of the sample diameter distributions were also the smallest for this method, indicating the highest uniformity of GUV size compared to the other two protocols (Figure 2c). The advantage of depositing MLVs instead of LUVs is the shorter protocol duration, as the conversion to LUVs is skipped. However, the samples made from the MLV deposits displayed a lower reproducibility (Figure 2b) and a more heterogeneous GUV size distribution (Figure 2c) compared to samples obtained by depositing the LUV 100 vesicles. This could be due to the larger average diameter and polydispersity index of MLVs compared to those of LUVs, as smaller vesicles rupture more easily compared to larger ones. The average diameter of the GUVs was the largest when the LUV 50 vesicles were used. Since smaller vesicles rupture more easily, we expected the reproducibility to be the highest for the LUV 50 vesicles, but this was not the case (Figure 2c). This is probably a side effect of the additional extrusion compared to that of the LUV 100 vesicles. We tried to produce LUV 50 vesicles directly from MLVs, but the flow resistance was too great, especially at higher Chol concentrations. Consequently, we first produced LUV 100 vesicles and then passed them through a 50 nm filter, as LUV 100 vesicles offered less resistance compared to the MLVs. Due to the additional extrusion, the duration of the protocol was even longer, causing a greater loss of final suspension volume, as part of the lipid suspension is always lost during the extrusion process due to fluid leakage. Consequently, the GUV distributions we obtained after the deposition of such suspensions were less reproducible (Figure 2b,c), as a different total lipid amount was deposited on the electrode. We also tried extruding the vesicles through a 30 nm filter, but this only exacerbated the problem of high resistance, so we decided to exclude such suspensions from the comparison.

### 3.2. The Effect of Chol Content

**Figure 3:**
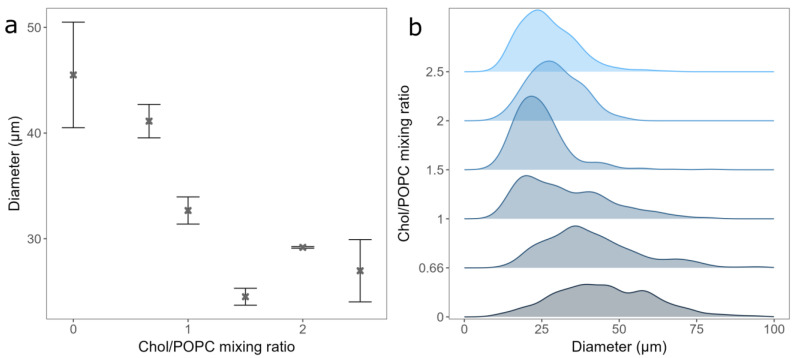
(**a**) GUVs mean diameters for different Chol/POPC mixing ratios. Points and bars represent mean values and their standard errors. (**b**) Size distribution densities of GUVs for different Chol/POPC mixing ratios. All experiments were performed using the deposition of LUV 100 vesicles spin-coated onto electrode for 30 s.

After deciding on the duration of spin-coating and the vesicle type to be used for deposition, we tested the effect of Chol content on GUV electroformation. The mean diameter and standard errors of the formed GUVs were 45.5 ± 5 µm, 41.13 ± 1.68 µm, 32.67 ± 1.28 µm, 24.51 ± 0.8 µm, 29.18 ± 0.08 µm, and 26.97 ± 2.95 µm, respectively, for a Chol/POPC mixing ratio between 0 and 2.5 (Figure 3a). As expected, the distribution of the vesicle diameters shifted to lower values with increasing Chol content (Figure 3b) [18]. This could be due to decreasing membrane elasticity and increasing rigidity when more Chol is added, making vesicle formation more difficult. For Chol/POPC mixing ratios greater than 1.5 (more than 60% of Chol in the mixture), where the GUVs may contain CBDs, a significant decrease in the average GUV diameter is measured. It is about 40% lower compared to that of the pure POPC bilayer.

For Chol/POPC mixing ratios of 2 and lower, we found only a couple of small anhydrous Chol crystals per sample. This is probably due to small dry patches forming on the electrode during spin-coating. However, compared to electroformation samples made using a completely dry lipid film, this amount was negligible.

### 3.3. Comparison with Samples Obtained from Completely Dry Lipid Films

**Figure 4:**
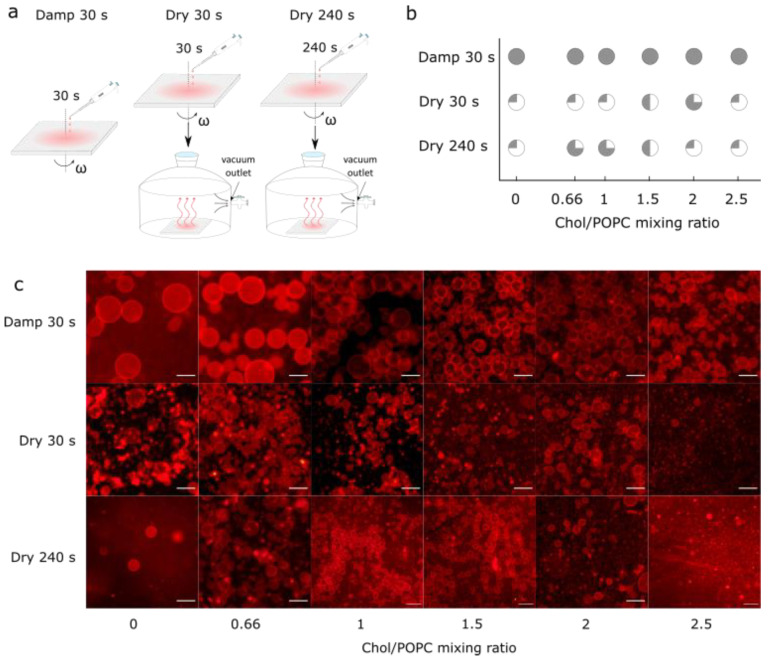
(**a**) Schematic depiction of differences obtained using our novel protocol for three different experiments. In the first experiment, damp lipid film was formed by depositing LUV 100 vesicles on an electrode and spinning it for 30 s at 600 rpm angular velocity (Damp 30 s). In the second experiment, Damp 30 s lipid film was held under vacuum for 30 min (Dry 30 s). In the third experiment, instead of spinning for 30 s, the electrode was spun for 240 s, and afterwards held under vacuum for 30 min (Dry 240 s). (**b**) Success of electroformation using Damp 30 s, Dry 30 s, and Dry 240 s protocols for obtaining lipid films, depending on different Chol/POPC mixing ratios. Success is based on the population homogeneity, yield, and number of defects. It is displayed through circle fullness, where a fuller circle indicates greater success. Empty circles (white circle) denote that no GUVs were formed or that their number was negligible; quarter circles represent low, half and three-quarter circles indicate medium, and full circles (gray circle) indicate high success. (**c**) Fluorescence microscopy images of GUVs for the different Chol/POPC mixing ratios using three different approaches for obtaining lipid films. The scale bar represents 50 µm.

Finally, the samples prepared using the optimized protocol were compared with those obtained from fully dried lipid films. These additional samples were made by spin-coating the aqueous lipid suspension for either 30 s or 240 s and then placing the coated electrodes in a desiccator for 30 min to ensure that any residual water was removed from the sample (Figure 4a). Drying not only increased the number of Chol crystals, but also decreased the GUV yield (Figure 4b,c). At a low Chol content, a negligible number of GUVs were found in the samples. For the high Chol content, the GUV yield was much lower compared to that obtained in the equivalent damp lipid film scenario, with many defects and anhydrous Chol crystals.

## 4. Conclusions

In this study, we optimized an improved electroformation protocol that bypasses the dry lipid film phase of the traditional electroformation method and combines the RSE method, plasma cleaning, and spin-coating techniques to obtain a damp lipid film. To further optimize the protocol, we conducted additional experiments to test the effect of using different vesicle types during lipid film deposition, the effect of spin-coating duration, and the effect of different Chol concentrations. In order to confirm the utility of the optimized protocol, our samples were also compared to those obtained from fully dried lipid films.

A spin-coating duration of 30 s was found to be optimal in terms of the balance between electroformation successfulness and lipid film dampness. A longer spin-coating duration would dry out the lipid film and exacerbate Chol demixing artifact. Compared to previous protocols that used damp lipid films, such as that employed by Baykal-Caglar et al., our method significantly shortened the preparation time by eliminating the 22–25 h high-humidity drying phase and replacing it with 30 s of spin-coating [3].

In terms of the vesicle type used for film deposition, the LUV 100 vesicles provided the best results in terms of reproducibility, even though the average diameter was smaller compared to that of the MLVs and the LUV 50 vesicles. The advantage of depositing MLVs instead of LUVs is the shorter protocol duration. However, samples made from MLV deposits displayed lower reproducibility and a more heterogeneous GUV size distribution. This is probably due to a larger average diameter and polydispersity index of the MLVs compared to the LUVs, as smaller vesicles rupture more easily compared to larger ones. The average diameter of the GUVs was the greatest when the LUV 50 vesicles were used, but the reproducibility of the samples was the lowest. This is probably a side effect of the additional extrusion performed compared to that used for the LUV 100 vesicles. Due to high flow resistance through a 50 nm filter, we could not directly extrude MLVs. We first produced LUV 100 vesicles and then passed them through a 50 nm filter. The additional extrusion step extends the duration of the protocol even further and causes a greater loss of final suspension volume, as part of the lipid suspension is always lost through liquid leakage during the extrusion process. This problem was even greater in our case, as the resistance increased along with the increasing Chol/POPC mixing ratio.

Finally, our samples were also compared with those obtained from fully dried lipid films. Drying increased the amount of Chol crystals found, which proves that the lipid film should never be subjected to a drying phase when using mixtures with a high Chol content.

This protocol allows us to successfully prepare GUVs and study the physical properties, lateral organization, and domain function of lipid membranes with a very high Chol content, such as the fiber cell plasma membrane of the eye lens. Furthermore, the avoidance of organic solutions and plasma cleaning of the electrode have been shown to be advantageous for the preparation of GUVs containing charged lipids and buffer solutions. Consequently, we believe that our protocol might also prove successful in those cases as well. Additionally, the protocol could also be adapted for protein–membrane interaction studies because protein denaturation is reduced by avoiding the dry film phase and due to the absence of organic solvents [11,12,13].

## Data Availability

The data presented in this study are available upon reasonable request from the corresponding author.
